# Within-host and between-host evolutionary rates across the HIV-1 genome

**DOI:** 10.1186/1742-4690-10-49

**Published:** 2013-05-02

**Authors:** Samuel Alizon, Christophe Fraser

**Affiliations:** 1 Laboratoire MIVEGEC (UMR CNRS 5290, IRD 224, UM1, UM2), 911 avenue Agropolis, B.P. 64501, 34394 Montpellier Cedex 5, France; 2 Medical Research Council Centre for Outbreak Modelling and Analysis, Department of Infectious Disease Epidemiology, School of Public Health, Imperial College London, St. Mary’s Campus, London W2 1PG, UK

## Abstract

**Background:**

HIV evolves rapidly at the epidemiological level but also at the within-host level. The virus’ within-host evolutionary rates have been argued to be much higher than its between-host evolutionary rates. However, this conclusion relies on analyses of a short portion of the virus envelope gene. Here, we study in detail these evolutionary rates across the HIV genome.

**Results:**

We build phylogenies using a relaxed molecular clock assumption to estimate evolutionary rates in different regions of the HIV genome. We find that these rates vary strongly across the genome, with higher rates in the envelope gene (*env*). Within-host evolutionary rates are consistently higher than between-host rates throughout the HIV genome. This difference is significantly more pronounced in *env*. Finally, we find weak differences between overlapping and non-overlapping regions.

**Conclusions:**

We provide a genome-wide overview of the differences in the HIV rates of molecular evolution at the within- and between-host levels. Contrary to hepatitis C virus, where differences are only located in the envelope gene, within-host evolutionary rates are higher than between-host evolutionary rates across the whole HIV genome. This supports the hypothesis that HIV strains that are less adapted to the host have an advantage during transmission. The most likely mechanism for this is storage and then preferential transmission of viruses in latent T-cells. These results shed a new light on the role of the transmission bottleneck in the evolutionary dynamics of HIV.

## Background

HIV evolves rapidly over the course of an infection due to its short generation time and to the selective pressure exerted by the host’s immune response [1,2]. The virus is therefore subject to multi-level selective pressures: at the within-host level, natural selection favours virus strains that grow rapidly inside the host and/or that escape the immune response, whereas at the between-host level it favours strains that spread rapidly in the host population. Within-host and between-host selective pressures can be conflicting as mutations that confer adaptation to exploit one host can impede the transmission rate to other hosts or can even be detrimental in another host [3]. Understanding the interplay between these levels of selection is fundamental to developing epidemiological models for the spread of drug resistant and immune escape mutants [4,5]. Here, we estimate HIV evolutionary rates at the within-host (WH) and between-host (BH) levels, and across the HIV-1 genome.

If all HIV strains inside an infected individual are equally likely to be transmitted to another host, evolutionary rates should have similar values at the WH and BH levels. On the contrary, current (but limited) evidence suggests that BH rates are lower than WH rates by an order of magnitude: the former tend to be close to 10^-2^ substitutions per site per year (subst ·*site*^-1^·*year*^-1^), whereas the latter are closer to 10^-3 ^subst ·*site*^-1^·*year*^-1^[4,6]. However, this conclusion is based on only a portion of the envelope (*env*) gene (using data from [1]) and evidence obtained on hepatitis C virus shows that different regions of the genome can evolve differently WH and BH [7].

We focus on the virus molecular rate of evolution, i.e. the number of mutations that are fixated per unit of time in the virus population. This substitution rate indicates the evolutionary potential of a population and is often referred to as the ‘evolutionary rate’ (ER). Importantly, the ER should not be confused with the mutation rate [8], which is the rate at which mutational errors occur during genome replication: the ER is a property of a viral population and is the result of evolutionary processes such as natural selection or drift, whereas the mutation rate is the result of the interaction between a virus and a host cell. The ER can be measured at the within-host level, by collecting longitudinal sequence data from the same infected host, but also at the between-host level by collecting sequence data from different hosts. Technically, we estimate the ER by assuming a relaxed molecular clock [9], when building the phylogeny using Bayesian inference methods [10]. This allows us to alleviate the limiting assumption that evolutionary rates are constant among lineages of the phylogeny and through time, i.e. the strict molecular clock hypothesis [8]. Note that we do test that the relaxed molecular clock assumption explains the sequence data better than the strict clock assumption (see the Methods).

**Figure 1:**
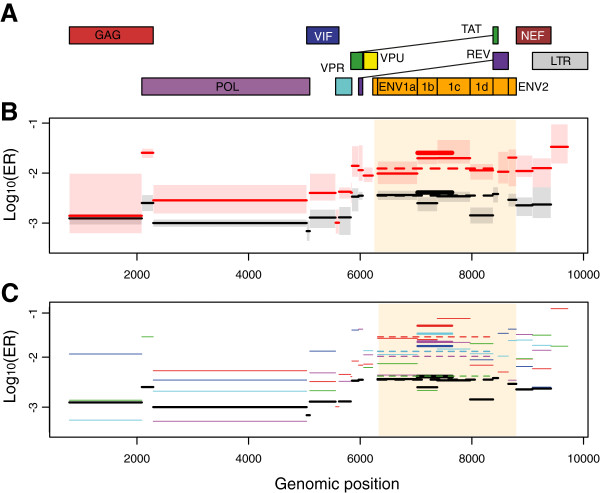
**Evolutionary rates (ER) throughout the HIV genome within- and between-hosts. A**) HIV genome, **B**) Median evolutionary rates for the pooled WH data (in red) and BH data (in black) and **C**) Median evolutionary rates for all the datasets. In panel B, shaded boxes indicate 50% credibility intervals. The thick line shows the C2V5 region (studied by former studies) and the dashed line the ENV1 segment. The *env *gene is highlighted in yellow. In panel C, the colour code is red for PIC1362, green for PIC38417, blue for PIC71101, cyan for PIC83747, purple for PIC90770 and black for the BH data (UP-up4).

We used full-length genomes from the Los Alamos National HIV Database. For the WH level, the database contained one adequate dataset, originating from a US patient [11,12]. Four additional datasets [13], also from US patients, could be analysed but they were all from acute infections (they spanned at most over 11 months) and had lower genome-wide coverage. For the BH data, many of the sequences we used originated from a study conducted in Boston [14]. In order to investigate evolutionary rates across the HIV genome, we had to consider specific sub-regions of the genome separately. We proceeded by splitting the genome into segments according to regions of overlaps between reading frames (Figure 1A). This also allowed us to also investigate the difference in evolutionary rates between overlapping and non-overlapping regions. Note that other WH and BH datasets were used to investigate evolutionary rates in a specific part of the genome (the *pol* gene) as discussed in the Results and discussion and in the Methods.

Our assumption of a relaxed molecular clock allowed us to estimate ER on internal and on external branches of the virus phylogeny separately [15]. At the WH level, we know that internal branches correspond to viruses that will have an offspring. For external branches however, this is not always the case. Therefore, we expect some of the viruses sampled to bear more deleterious mutations in their genome. In other words, at the WH level, we can expect the substitution rate on external branches to be higher and closer to the virus mutation rate. At the BH level, we do not expect much differences between ER on internal and external branches because selection has had the time to act.

Concerning HIV, it is known that BH substitution rates in the *env *gene are higher than that in the *gag *gene [16]. We are not aware of studies that compare WH and BH evolutionary rates in different genomic regions. Here, we provide a genome-wide overview of molecular rates of evolution of HIV-1 both at the within- and at the between-host levels.

## Results and discussion

### Presence of molecular signal

As stressed by several studies [8,9], before analysing evolutionary rates, it is necessary to check that there is actually molecular clock signal in the data, i.e. that there is accumulation of sequence divergence through time and that this temporal signal is not too over-dispersed. This can be done in several ways, which are further described in the Methods.

**Figure 2:**
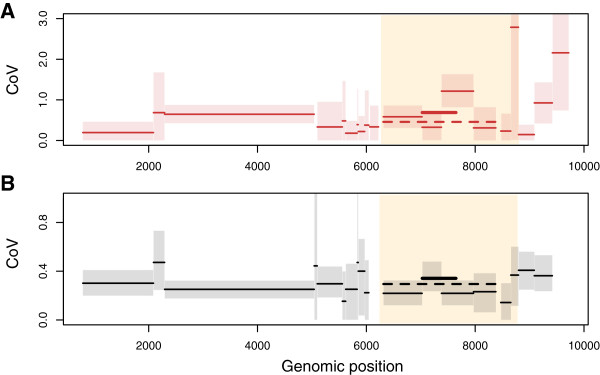
**Coefficient of variation statistics (scaled variance in evolutionary rate among lineages). A**) In patient PIC1362 and **B**) in the BH dataset US-up4. Shaded boxes indicate the range of the 95% Highest Posterior Density (HPD). The thick line shows the C2V5 region (studied by former studies) and the dashed line the ENV1 segment. The *env *gene is highlighted in yellow.

First, we looked at the coefficient of variation statistics (CoV), i.e. the scaled variance in ER among lineages [9], which was obtained when inferring phylogenies using Bayesian methods. As mentioned in the Methods section, this tests the over-dispersion of the molecular clock signal. If the posterior distribution of the CoV does not impinge substantially on the boundary at zero, it supports the relaxed molecular clock model. At the BH level, the coefficient of variation (CoV) of the ER did not strongly vary across the HIV genome: it was always close to 0.3 (Figure 2B), which is consistent with earlier results obtained in the C2V5 region only [4]. At the WH level, in patient PIC1362 the CoV were higher and more variable (Figure 2A). This discrepancy between WH and BH estimates of CoV is consistent with what has been reported in the hepatitis C genome [7]. In the four other WH datasets from acute infections, on average the CoV were more similar to that observed at the BH level (Additional file 1: Figure S3), but this was likely caused by the fact that several segments had lower confidence interval very close to 0 (and were ignored in subsequent analyses).

The second method, which tests for the temporal signal (i.e. evidence for the accumulation of sequence divergence), consists in performing a regression between root-to-tip divergence and sampling date in a ‘classical’ phylogenetic tree (with a strict molecular clock assumption). The *R*^2 ^of the regression indicates the amount of molecular clock signal and we refer to it as the ‘root-to-tip’ method. We found that the WH sequences seemed to exhibit more signal than the BH host sequences, especially in the *env *region (Additional file 1: Figure S1).

We also used a third method, which tests for the temporal signal by randomising tip dates. We only applied this method to the C2V5 region for computational reasons and detected significant molecular clock signal (Additional file 1: Figure S2). Finally, the comparisons we performed between the likelihood of the strict clock model and the relaxed molecular clock models offers another way to test for the adequacy of the model.

Some segments exhibited weak molecular clock signal using both the CoV and the root-to-tip methods and were removed from the statistical analyses. At the BH level, the three (out of 21) segments ignored were REV-ENV, VIF-VPR and VPR-TAT, which altogether represent 2.3% of the total sequence length considered (see Additional file 2: Table S2 for the complete list of segments). At the WH level, the segments ignored for PIC1392 were ENV2, GAG, GAG-POL, TAT-REV-ENV and POL-VIF (i.e. 15.4% of the total sequence length). In the other four WH datasets, we removed segments C2V5, ENV1–3, ENV2, POL, POL-VIF, REV-ENV, TAT, TAT-REV, TAT-REV-ENV, VIF-VPR, VPR and VPR-TAT in PIC38417 (i.e. 42.4% of the total sequence length), segments ENV1–1, ENV1–3, LTR3, GAG-POL, POL-VIF, TAT-REV, TAT-REV-ENV, VIF-VPR, VPR, VPR-TAT and VPU in PIC71101 (i.e. 21.4% of the total sequence length), segments GAG-POL, POL-VIF, TAT, TAT-REV, TAT-REV-ENV, VIF, VIF-VPR, VPR-TAT, VPR and VPU-ENV in PIC83747 (i.e. 11.2% of the total sequence length) and segments ENV1–4, GAG, GAG-POL, LTR3, POL-VIF, REV-ENV, TAT, TAT-REV-ENV, VIF-VPR and VPU-ENV in PIC90770 (i.e. 14.3% of the total sequence length). The fact that many of these segments belong to overlapping reading frames is discussed below. Overall, dataset PIC1362 was our WH dataset with the best coverage (see Additional file 2: Figure S1). In the following we use it as our reference dataset to stress some specific points.

### Comparing rates on internal and external branches

**Figure 3:**
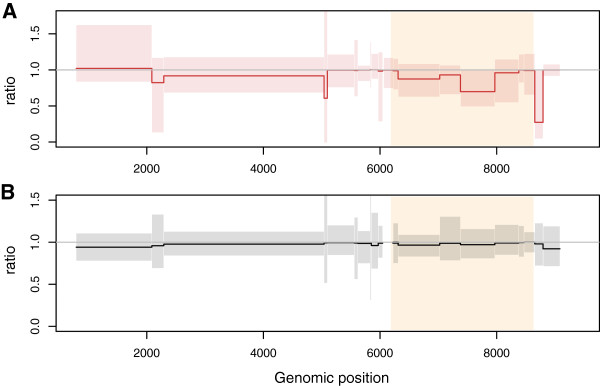
**Ratio between the substitution rate estimated on internal branches and that estimated on external branches. A**) In patient PIC1362 and **B**) in the BH dataset US-up4. The grey horizontal line indicates equal evolutionary rates on internal and external branches. Shaded boxes indicate the 95% confidence interval. The *env *gene is highlighted in yellow.

At the WH level, especially in the PIC1362 dataset, we found a mismatch between ER measured on internal and external branches of the phylogeny, with lower rates on the internal branches (Figure 3A). This is not surprising since, as mentioned earlier, selection has had little time to act and some of the virus sequences sampled could contain deleterious mutations. Note that the ratio between ER on the internal and on the external branches is particularly low in *env*, which is the only region considered by earlier studies [4,6]. On the contrary, at the BH level, this ratio is always close to 1 suggesting that evolutionary rates are more homogeneous on the phylogeny (Figure 3B). Fitted values for each genomic segment are shown in Additional file 2: Table S3.

In the other WH datasets, the values of these ratios were much more dispersed. This could be due to the fact that these data come from acute infections.

In the following, in order to compare substitution rates at the WH and at the BH level, we only use rates estimated from internal branches of the phylogeny (otherwise, the fact that the WH evolutionary rate is closer to a mutation rate would bias the analyses).

### Evolutionary rates

Evolutionary rates varied across the HIV genome (Figure 1B) and were significantly higher in the *env *region than in the rest of the genome, both at the WH and at the BH levels (Table 1). Figure 1C shows the evolutionary rates for all the datasets pooled in Figure 1B (see also Figure S4 in Additional file 1 for more detailed results on all the datasets and Table S2 in Additional file 2 for the exact ER values in each segment).

**Table 1:** **Effect of level of study (WH or BH) and of the presence or not in the *env * gene on evolutionary rates in PIC1362 and US-up4**

	**log**_**10 **_**(WHER)**	**log**_**10 **_**(BHER)**	**p-value**
ENV	-1.84 (-2.78,-1.28)	-2.51 (-3.38,-2.01)	^∗∗∗^
Non-ENV	-2.12 (-3.48,-1.21)	-2.78 (-3.70,-2.17)	^∗∗∗^
p-value	^∗∗∗^	^∗∗∗^	

The cells contain median values with 95% confidence intervals. P-values indicate the result of the outcome of a two-tailed t-test (‘ ^∗∗∗^’ indicates a p-value lower than 0.001, ‘ ^∗∗^’ a p-value lower than 0.01 and ‘ ^∗^’ a p-value lower than 0.05).

**Figure 4:**
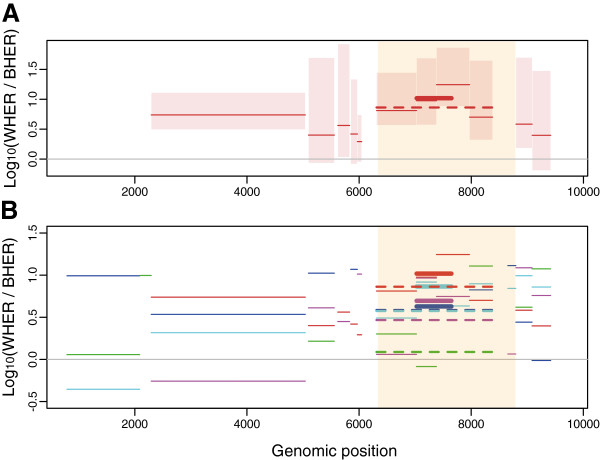
**Log of the ratio between the median ER measured WH and BH. A**) In patient PIC1362 and **B**) for all the datasets. In panel A, the boxes are the 95% confidence interval. The thick line indicates the C2V5 segment (studied by earlier studies) and the thick dashed line indicates the whole ENV1 segment. In panel B, the colour code is the same as in Figure 3. In the *env* gene (the shaded yellow area) the ratio between WH ER and BH ER is significantly higher.

Furthermore, WH evolutionary rates were significantly higher than BH evolutionary rates. This can be seen in Table 1, which reveals a 4.7 fold difference (0.67 log_10_) in the *env* gene and a 4.6 fold difference (0.66 log_10_) elsewhere in the genome for the pooled data. When we focus on patient PIC1362, this difference in ER in *env *(shaded area in Figure 4) is clearer (5.75 fold difference in *env *vs. 3.98 elsewhere in the genome). We also found a significant difference when we compared the ratio between WH ER and BH ER observed in *env *to that observed in the rest of the genome ( *t *= 17.3, df = 2035.7, p-value < 10^-3^).

We also considered the ‘Overlap’ factor, i.e. whether or not the segment is an overlapping reading frame. As expected, in the WH dataset, we found lower rates in overlapping regions (factor 1.3, see Table 2). For the BH dataset, the difference was slightly less important (factor 1.2) and even went in the opposite direction. Many of the segments in overlapping reading frames also exhibited so little molecular clock signal that an ER could not be estimated at all (see the list of segments excluded above), further suggesting that mutations are especially likely to be deleterious in these regions of the viral genome. Overall, care should be taken when interpreting this result due to the high number of overlapping segments that had to be removed in the analysis.

**Table 2:** Effect of level of study (WH or BH) and the overlap factors on evolutionary rates

	**log**_**10 **_**(WHER)**	**log**_**10 **_**(BHER)**	**p-value**
Non-Overlapping	-1.94 (-3.31,-1.22)	-2.70 (-3.58,-2.21)	^∗∗∗^
Overlapping	-2.05 (-3.21,-1.33)	-2.61 (-3.70,-1.92)	^∗∗∗^
p-value	^∗∗^	^∗∗^	

There is an interaction between the level and the overlap: overlapping regions evolve more rapidly at the BH level but less rapidly at the WH level. P-value notations are identical to Table 1.

In order to check for the robustness of our substitution model assumption, we also measured these evolutionary rates on phylogenies inferred using a GTR substitution model (instead of a HKY+ *Γ *model). As shown in Additional file 1 (Figure S5), the substitution model did not seem to affect the results qualitatively but the HKY model yielded slightly higher estimates for the ER, both at the WH and at the BH levels.

### Other datasets

WH longitudinal data appropriate for these types of analyses are rare. We analysed several whole-genome longitudinal data (patient 9213 studied in [17] and four patients –PIC38417, PIC71101, PIC83747 and PIC90770– studied in [13]) but none of the data matched that of patient PIC1362. Overall, the molecular clock signal (estimated using the root-to-tip divergence method and the coefficient of variation method) was low in many of the segments (see above for the list of the segments removed). Furthermore, in patient 9213, almost none of the phylogenies converged in BEAST but in the few segments that did converge (e.g. VPR-TAT), results were consistent with that obtained in PIC1362 (results not shown).

As mentioned above, our results are consistent with earlier studies that have shown a significant difference in evolutionary rates in part of the *env* gene [6]. To further investigate the robustness of our results, we estimated evolutionary rates in part of the *pol *genes for other WH and BH datasets.

**Figure 5:**
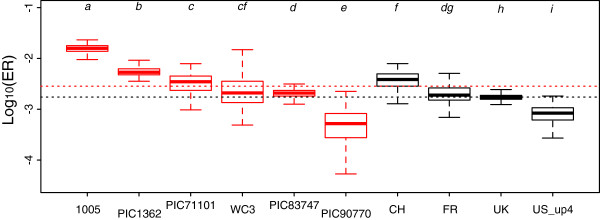
**Evolutionary rates (ER) in the *pol * gene within- and between-hosts in 10 datasets. **Within-host evolutionary rates are in red. The letters above the boxes indicate significant differences between datasets (t.test with a p-value <0.001). The dashed lines indicate the median ER of the WH data (in red) and of the BH data (in black) and these are significantly different (Welch two sample t-test, *t *= 17.3, df = 1418, p-val. < 0.001).

Estimates of WH and BH evolutionary rates from the POL region for 6 WH datasets and 4 BH datasets supported the differences in ER reported above (Figure 5): the median ER at the WH level was 2.85 · 10^-3 ^*vs. *1.74 · 10^-3 ^at the BH level (a significant 1.64 fold difference, *t *= 17.3, d.f. = 1417.7, p-value < 10^-4^). Figure 5 also illustrates that there can be great variations amongst patients or amongst BH datasets. Overall, only one WH dataset stood out (PIC90770) with an ER lower than all the ER measured at the BH level. However, the ER measured in other regions of the genome for this patient were not lower than expected (Additional file 1: Figure S4). This illustrates the utility of working with whole virus genomes originating from the same patient.

These additional results show that estimating within-host evolutionary rates requires detailed datasets that span over several years, with several sequences per time step. Appropriate data that is publicly available is limited but it is likely that there exist private datasets from which further insight could be gained.

### Discussion

HIV evolves during the course of an infection and adapts to its host. However, this evolution is ‘short-sighted’ in that it is unlikely to favour genotypes that are efficient at transmitting to new hosts. The hypothesis that there is a conflict between selective pressures acting on HIV at the WH and BH level is not new [3]. However, it has regained interest with more recent analyses of a portion of the HIV genome (located in the *env *gene of the virus), which found that substitution rates seem to be much higher at the WH level than at the BH level [4,6].

Here, we show that differences between WH and BH substitution rates previously observed in *env *are actually present throughout the whole genome. More precisely, the substitution rates do vary across genomic regions (with higher rates in *env*) but a difference of approximately one order of magnitude is nevertheless observed between the WH and the BH rates. This pattern supports the hypothesis that some HIV variants are stored early in the infection in latent cells and preferentially transmitted when re-activated later on [18]. Indeed, it is more parsimonious to assume that a virus is stored for several generations rather than assuming that there would be reverse mutations throughout the whole genome.

We found that the difference between WH and BH evolutionary rates was slightly (but significantly) more pronounced in the envelope gene (*env*). This suggests that another process could be at play in *env*, namely that some of the mutations acquired in this genomic region reverse rapidly in the early stage of an infection, which supports earlier results [13]. It is noteworthy that while longitudinal analysis of early evolution in whole genomes supports some reversion to wild-type in *env*, it does not support sustained reversion throughout the genome [13].

We have not considered synonymous and non-synonymous mutations explicitly. The main reason for this is that since we are carrying a whole-genome analysis, we include many regions of the HIV genome that have overlapping reading frame (in which there are no synonymous substitutions). Furthermore, codon usage bias is high for HIV [19] and, because of secondary and tertiary RNA structure, many synonymous mutations may turn out to be non-synonymous. However, we can still draw some conclusions from our results because we partitioned the genome according to regions of overlap, i.e. regions that simultaneously code for multiple genes. As expected, we found significantly lower rates in overlapping regions at the WH level (and in some cases, no evidence of clock-like evolution at all). At the BH level, this difference seemed to go in the other direction, which could be explained by more time for negative selection to act on deleterious mutations at the BH level. This would be consistent with the absence of differences in BH rates between internal and external branches but these results would require more data (especially BH data) to be confirmed.

Results shown in earlier reviews (though never described in depth) [4,6] have stimulated research on virus evolution at different levels. However, it is difficult to compare these results to ours because, due to lack of space, their authors did not describe the protocol they used. For instance, we do not know which substitution model they used or, more importantly, on which type of branches (all branches or internal branches only) they measured the evolutionary rates.

We are only aware of one other study that compared WH and BH evolutionary rates across a whole virus genome [7]. This other study was conducted on hepatitis C virus (HCV). Since HCV has no overlapping reading frames, the authors could cut the virus genome into segments of similar size. The main difference between their study and ours is that the WH evolutionary rates were estimated by pooling data from 15 different individuals, who were all infected by the same source via blood transfusion. Our results corroborate these results on HCV in that evolutionary rates vary across the genome and that the difference between WH and BH evolutionary rates is more pronounced in the envelope region. However, contrary to HCV, there is a difference between WH and BH rates even outside *env*, which allows us to hypothesise that the nature of transmitted strains differ for these two viruses.

A notable limitation to the generality of our results is that we were only able to analyse full genome sequence data of few patients to estimate WH rates. In order to generalise these results, one should analyse more WH genomes (preferentially sampled from patients with infections progressing at different rates).

Overall, this illustrates that estimating within-host evolutionary rates requires extremely good quality datasets that have both a long longitudinal coverage and a deep sampling at each time point. This limitation is not technical and in fact it might be that such data already exists. However, it is not publicly available so far.

## Conclusion

We show that evolutionary rates vary strongly across the HIV genome, with higher rates in the envelope gene (*env*). Furthermore, within-host evolutionary rates are consistently higher than between-host rate throughout the HIV genome. This difference is significantly more pronounced in *env*. While this result is based on the analysis of only one patient with a long time-series and four patients followed during acute infection and for a short period afterwards, it is an extension of a result that is already established from variation in *env *in several other patients. Finally, we find only weak differences between overlapping and non-overlapping regions. This study provides the first genome-wide overview of the differences in the HIV rates of molecular evolution at the within- and between-host levels. Contrary to hepatitis C virus, for which this difference is only located in the envelope gene, within-host evolutionary rates are higher than between-host evolutionary rates across the whole HIV genome. This supports the hypothesis that HIV strains that are less adapted to the host have an advantage during transmission. The most likely mechanism for this is storage and then preferential transmission of viruses in latent T-cells. These results shed a new light on the role of the transmission bottleneck in the evolutionary dynamics of HIV. Further studies involving more data (especially within-host data) are needed to determine how these results can be affected by host specificity.

## Methods

Estimating ER is a very popular approach but there are several pitfalls to avoid. The steps we followed (eventually, having fallen into many pits along the way) are highlighted in Table 3.

**Table 3:** Method steps

	
i	Cut the genome sequences into segments
ii	Remove recombining sequences
iii	Check for the existence of molecular clock signal in the data
iv	Balancing datasets (to maximise clock-likeness)
v	Select the most appropriate substitution model
vi	Compare molecular clock models and coalescent models
vii	Run the bayesian phylogeny inference package (BEAST)
viii	Analyse substitution rates on internal and external branches

### i) Cutting the genome into segments

Sequences were cut into HIV genes using the Gene Cutter algorithm (http://www.hiv.lanl.gov/content/sequence/GENE_CUTTER/cutter.html). These genes were checked using SeaView v.4.3.3 [26] and cut according to overlapping regions using the ape package in R v.2.14.2 [27].

### ii) Removing recombinant sequences

Each segment was analysed with 6 different methods to detect recombination using the RDP software [28]. According to the designer of RDP, any sequence where at least one of the methods detects recombination can be considered as a recombinant. We applied this criterion here (with a p-value of 0.05).

We did not find any evidence for recombination in the WH dataset. In the BH dataset, some sequences were recombinant and were removed.

### iii) Controlling for molecular clock signal

An important step before estimating evolutionary rates with a relaxed molecular clock is to check that there is actually molecular clock signal in the data. Indeed, software packages such as BEAST [10] will always provide the user with an estimate of substitution rate, even if there is no molecular clock signal in the data. The presence of such signal, i.e. the ‘clock-likeness’ of the data, can be checked in different ways. Here we present three of these.

First, we checked that the posterior distribution of the coefficient of variation statistics (CoV), i.e. the scaled variance in ER among lineages [9], does not impinge substantially on the boundary at zero, which is a way to test between relaxed and strict molecular clock models [7].

Second, we estimated the root-to-tip divergence [8]. This provided us with an R-squared of the regression between root-to-tip divergence that indicates the amount of sequence divergence explained by the sampling date. To do so, we first generated phylogenies using a ML likelihood approach (using the software PhyML v.3.0 [29]). We then estimated the clock-like behaviour of the data by performing a regression between root-to-tip distance in the ML tree and the date of sampling of each sequence using the software Path-O-Gen v1.3 [30]. Trees were rooted at the position that was likely to be the most compatible with the assumption of the molecular clock. This method estimated the amount of variation in genetic distances that can be explained by the sampling time.

Third, we built phylogenies using datasets with randomised sampling dates (in order to scramble the temporal structure) and then estimated the evolutionary rate (ER) on the C2V5 segment. If the difference between the substitution rate obtained on the real phylogeny and those obtained on the randomised phylogenies is significant, it supports the existence of a temporal structure [31].

### iv) Balancing datasets

In order to maximise the ‘clock-likeness’ of the data, it helps to have a balanced dataset, i.e. a similar number of sequences from each time point and as many time points as possible [20]. This was obtained by removing samples (randomly) from the most overrepresented time points. For the WH dataset, we kept up to 13 sequences for each time point and for the BH dataset up to 4 (these numbers were chosen to maximise the signal in the C2V5 segment).

### v) Determining the substitution model

The substitution model was chosen using the software jModelTest v.0.1 [32]. We selected the HKY+ *Γ *model, which had the advantage to often provide a good (if not the best) fit to the data without being too complicated (Additional file 2: Table S1). This model also has the advantage to allow for comparisons with other studies, such as [15].

Note that for the WH data, a GTR substitution model sometimes fitted the data better than an HKY model. However, as we show, our results were not influenced qualitatively by the substitution model.

### vi) Determining molecular clock and coalescent models

The model with a relaxed log-normal molecular clock and a Bayesian skyline coalescent model [33] was selected using a Bayes Factors criterion [7,34] in the C2V5 region. The Bayes Factor (BF) is based on the difference between the log marginal likelihoods of each model. The classical rule of thumb is that if the difference in Bayes Factors is greater than 3, this is positive evidence for a difference between the two models, and if it is greater than 10, this is strong evidence.

### vii) Building the phylogenies

Phylogenies were inferred using BEAST v.1.6.2 [10] with default parameters. Simulations were run until convergence (i.e. an effective sample size greater than 200 for all parameters) and the results were summarised using Tracer v.1.5.

Evolutionary rates were first estimated on each posterior tree distribution using Tracer. Approximately 10% of the output was used as a burn-in. We also estimated the coefficient of variation of the evolutionary rate for each region.

For each data set, there was one tree posterior distribution for each genome segment and each of these posterior distributions were inferred using two different substitution models (GTR and HKY+ *Γ*).

### viii) Analysing substitution rates on internal and external branches

Using a relaxed molecular clock in BEAST allows us to estimate ER on different parts of the phylogeny. We obtained final estimates of ER on internal and external branches for 200 trees from the posterior distribution using the program RateAnalyzer.jar [15]. Note that even though part of the trees of the posterior distribution (200) were used in RateAnalyzer.jar, the results obtained were consistent with that obtained with Tracer, which used the full posterior distributions.

## The data

We selected full genomes from untreated US patients infected by HIV-1 subtype B from the Los Alamos HIV database http://www.hiv.lanl.gov/. The GenBank accession numbers of all the sequences we used are provided in Additional file 3.

There was only one excellent quality longitudinal dataset that fitted our criteria (subject PIC1362, a homosexual caucasian male who refused treatment during the whole infection [11,12]). The dataset consisted of 65 full genome sequences with sampling dates ranging from 1998 to 2002. Earlier studies show that, within a constraint of subsampling sequences that have been collected at a set of distinct sampling times, having an equal number per distinct time is best to maximise the molecular clock signal [20], this is why here we kept up to 13 sequences from each time point and ended up with a dataset of 65 sequences.

The Los Alamos HIV database did contain two other studies with longitudinal data of full genome sequences. With one of these datasets [17], the analyses were largely unsuccessful, probably because of a lack of molecular signal. More precisely, this dataset was based on 11 sequences sampled from a German patient (patient 9213) from 2004 to 2008 at 4 different time points. Four datasets from the study by Herbeck et al. [13] could be analysed. A common feature of these sequences is that they were all obtained during the acute phase of the infection (the longest longitudinal timespan was 11 months). This means that the unit for the estimation of the evolutionary rates was months instead of years. The patient codes were PIC38417, PIC71101, PIC83747 and PIC90770.

Finally, we measured evolutionary rates in the POL segment (a coverage of at least 900 nucleotides between positions 2300 and 4000 of the HIV genome). This looser selection criterion allowed us to include data from two other studies: patient WC3 from a study by Kemal et al. [21] and patient 1005 from a study by Kearney et al. [22] (other patients were analysed in this study but there was no molecular signal in their sequence data, data not shown).

For the BH dataset, we applied the same selection criteria (sequences had to be from HIV-1 subtype B, sampled in drug naive individuals from the US, with known sampling dates). Many sequences came from a study conducted in Boston [14] and additional sequences came from other studies [23-25]. As for the WH dataset, we homogeneised our sampling by keeping up to 4 sequences from each time point and ended up with a dataset of 30 sequences with sampling dates ranging from 1985 to 2007.

As for the WH level, we analysed other BH datasets for the POL segment. We thus obtained 35 sequences from France, 16 sequences from Switzerland and 106 sequences from the UK, all from the Los Alamos HIV database.

## Competing interests

The authors declare that they have no competing interests.

## Authors’ contributions

SA and CF conceived the study, SA collected and analysed the data, SA and CF wrote the manuscript. All authors read and approved the final manuscript.

## Supplementary Material

Additional file 1 A file with supplementary figures. Click here for file

Additional file 2 A file with supplementary tables. Click here for file

Additional file 3 A LibreOffice table with accession numbers of all the sequences used. Click here for file
