# Supplementary material for: Within-host and between-host evolutionary rates across the HIV-1 genome
Source: Retrovirology. 2013 May 2;10:49. doi: 10.1186/1742-4690-10-49 (PMC3685529; doi:10.1186/1742-4690-10-49)
Supplement: Additional file 1 — A file with supplementary figures. [file 1742-4690-10-49-S1.pdf]

## Supplementary Figures

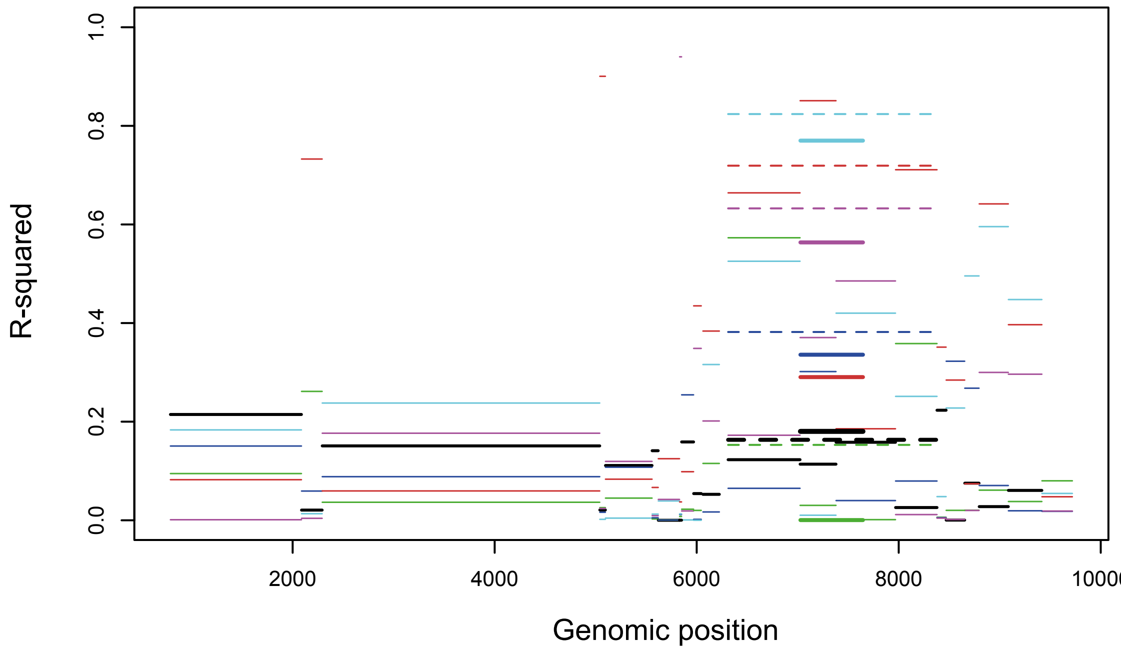


Figure S1: **R-squared of the root-to-tip divergence.** This measure is obtained using a strict molecular clock and the software Path-O-Gen. For further details, see the caption of Figure 1.

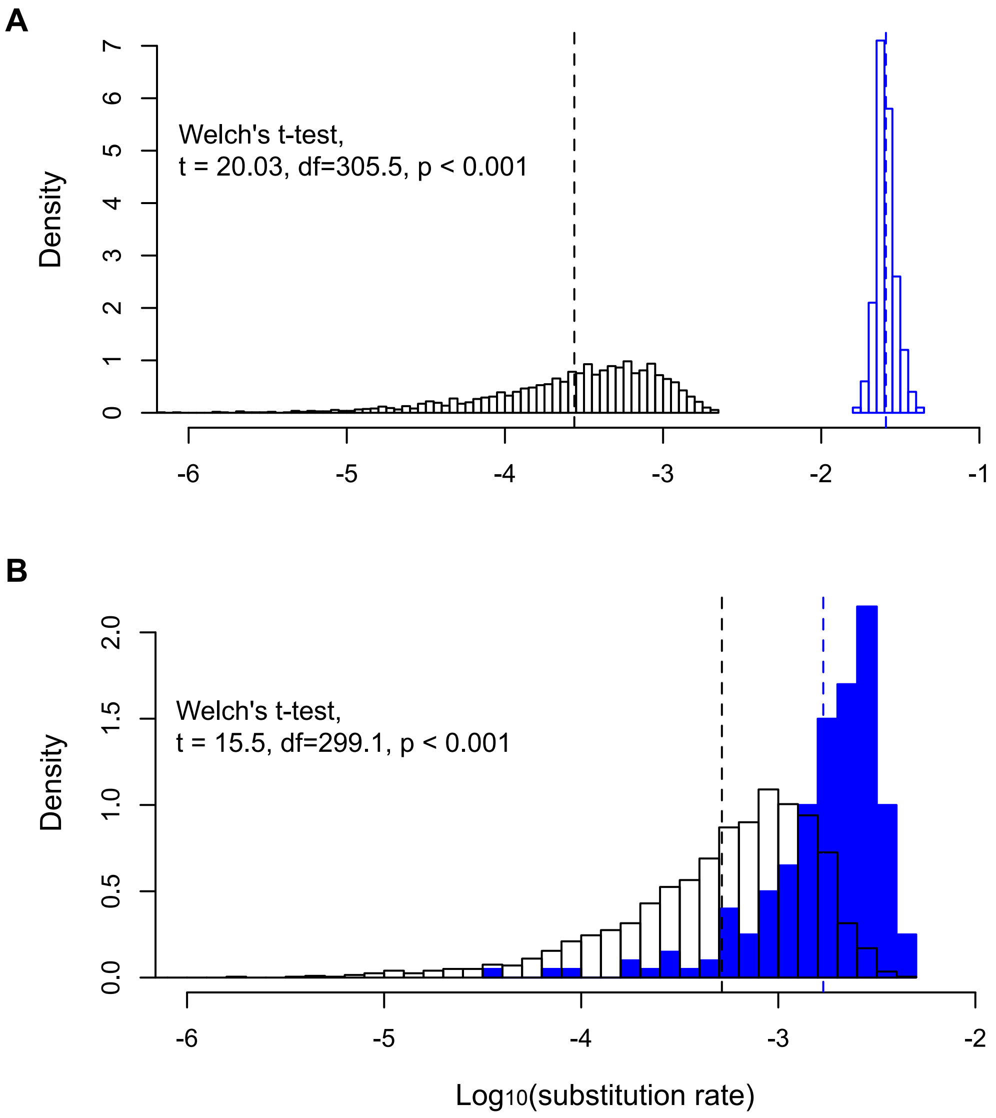


Figure S2: **Substitution rates in phylogenies with randomized dates (C2V5 segment) for the WH dataset (A) and the BH dataset (B).** For each dataset we generated 10 datasets with randomized tip dates. The posterior distribution of the ER obtained with the randomized phylogenies were compared to that obtained with the unaffected phylogeny (in blue). The ER from the randomized phylogenies are significantly lower, which supports the assumption of relaxed molecular clock.

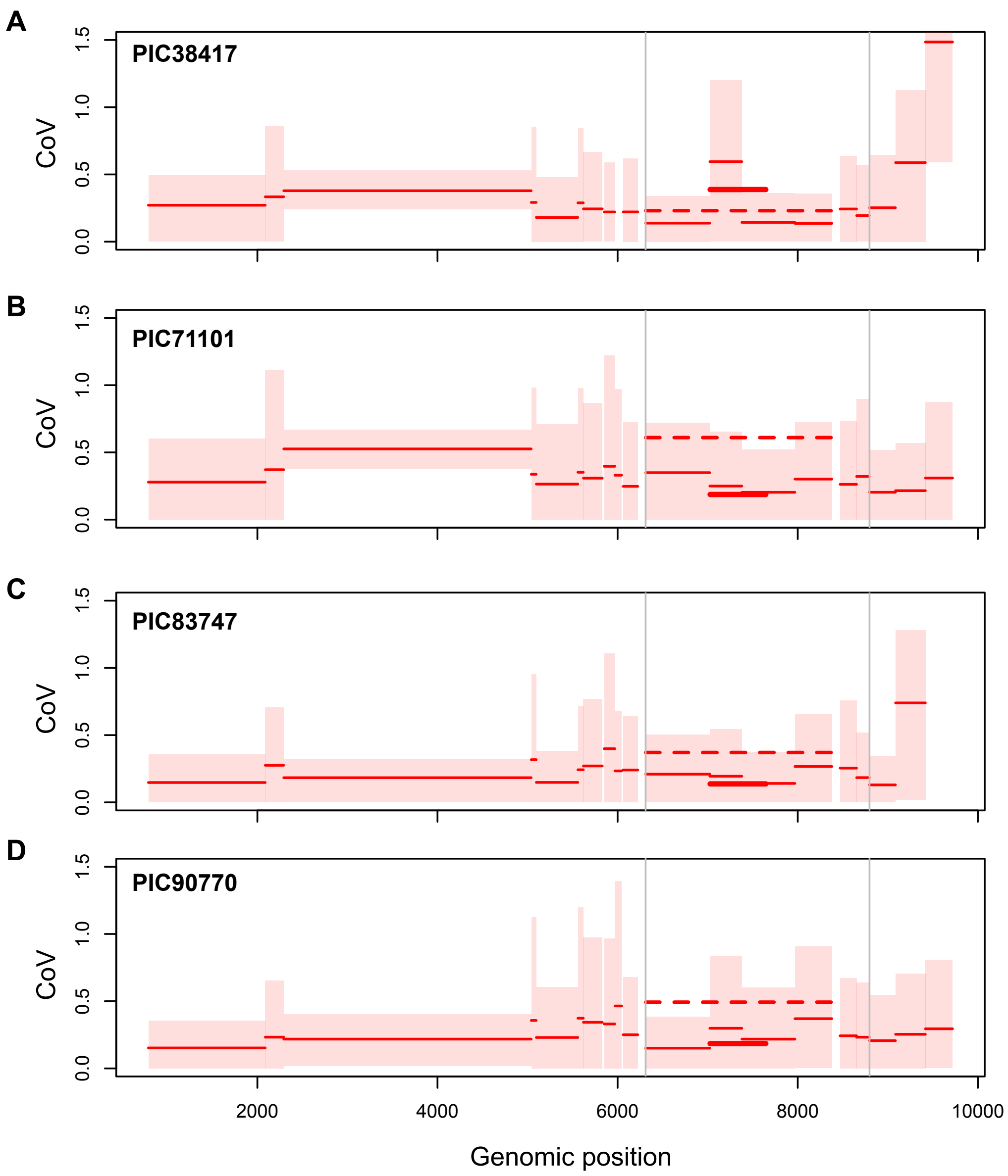


Figure S3: **Coefficient of variation statistics (scaled variance in evolutionary rate among lineages).** A) In patient PIC38417, B) in patient PIC71101, C) in patient PIC83747 and D) in patient PIC910770. Shaded boxes indicate the range of the 95 % Highest Posterior Density (HPD).

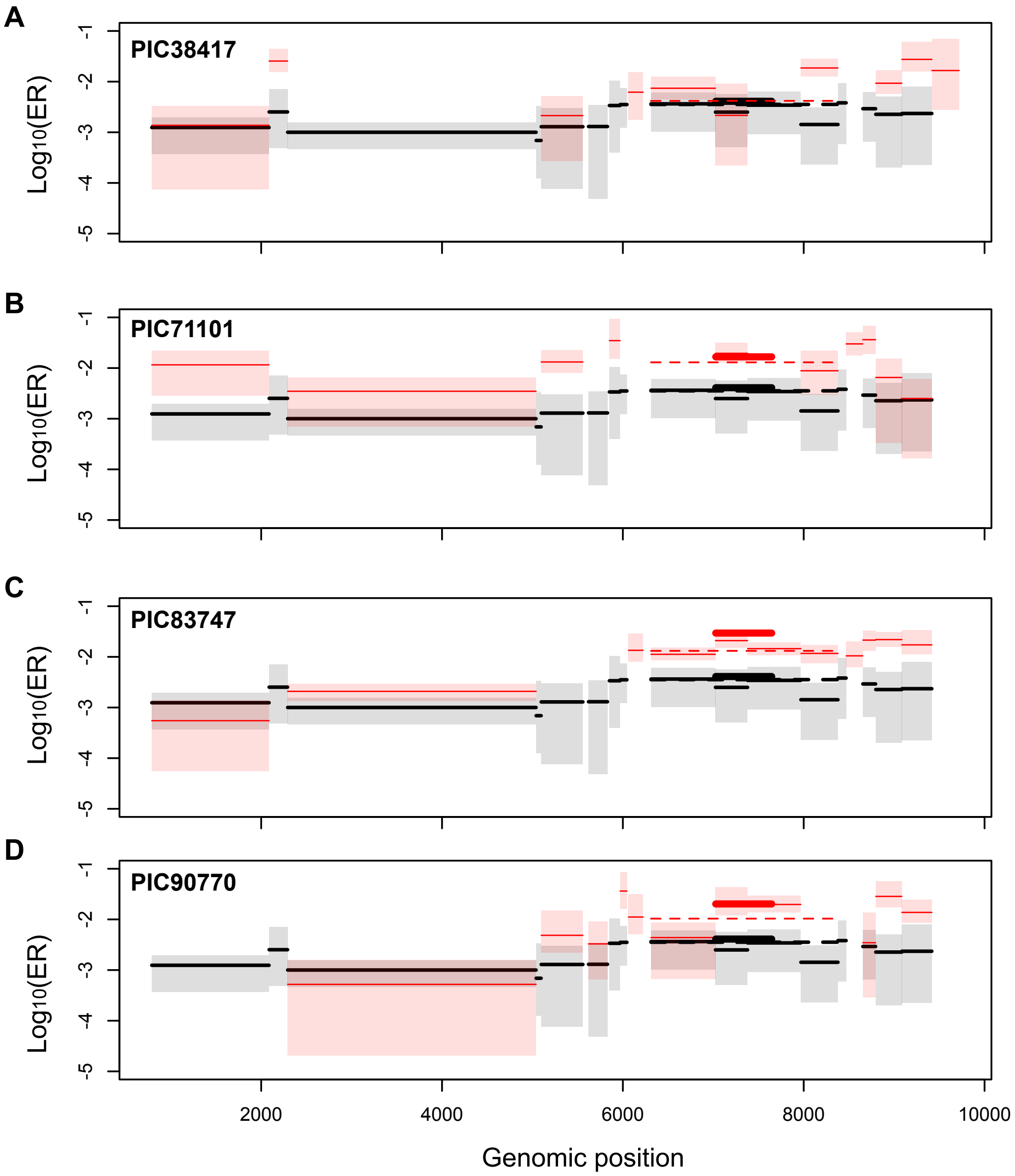


Figure S4: **Median evolutionary rates at the WH (in black) and BH level (in red)** A) In patient PIC38417, B) in patient PIC71101, C) in patient PIC83747 and D) in patient PIC910770. Shaded boxes indicate the range of the 95 % Highest Posterior Density (HPD). The thick line shows the C2V5 region (studied by former studies) and the dashed line the ENV1 region.

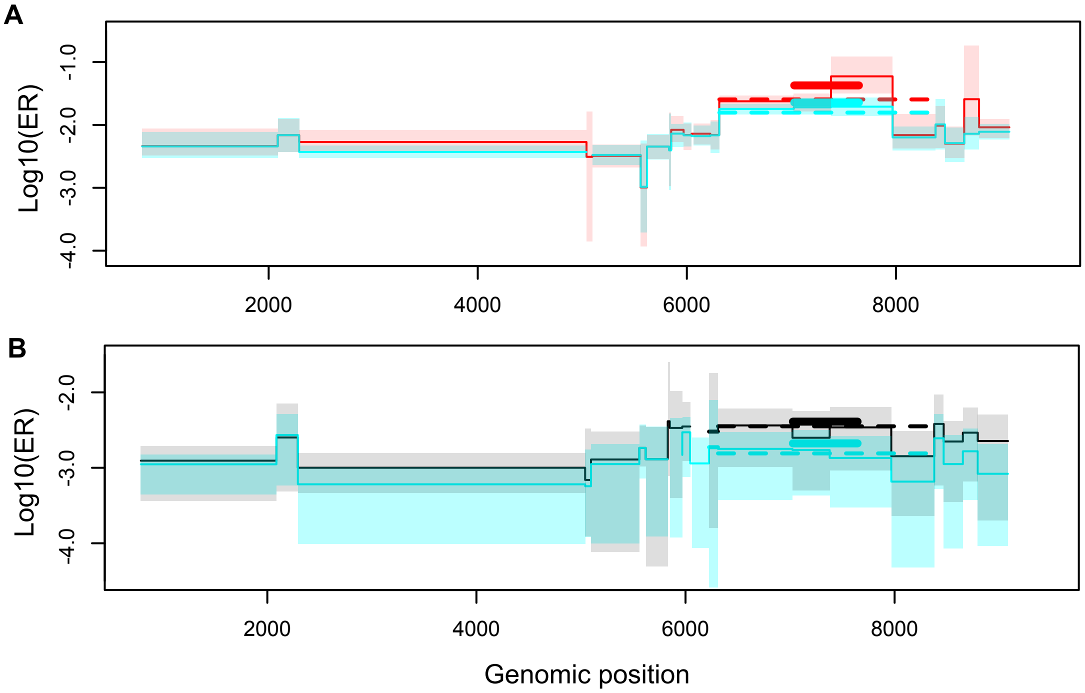


Figure S5: **Substitution rates in phylogenies built using a GTR model (in cyan) instead of the HKY+Γ model.** A) In patient PIC1362 and B) in the BH dataset US-up4. Shaded boxes indicate the 95 % confidence interval.
