# Supplementary material for: Within-host and between-host evolutionary rates across the HIV-1 genome
Source: Retrovirology. 2013 May 2;10:49. doi: 10.1186/1742-4690-10-49 (PMC3685529; doi:10.1186/1742-4690-10-49)
Supplement: Additional file 2 — A file with supplementary tables. [file 1742-4690-10-49-S2.pdf]

Supplementary Tables

Table S1: **Model Selection Analysis with an HKY+Γ substitution model.** For the molecular clock model, 'Relaxed' indicates an Uncorrelated Lognormal relaxed molecular clock. For the coalescent model, 'Constant' stands for constant population size, 'Expo' for exponential growth and 'BSP' for Bayesian skyline plot. 'ΔBF' is the difference in Bayes Factor with the best model using the method from [1] as implemented in Beast. Classically, ΔBF>3 is positive evidence and a ΔBF>10 is strong evidence for a difference between two models.

Data set	Molecular clock model	Coalescent model	Marginal Log Likelihood	Genomic rate of evolution ($\times 10^{-2}$)	Relaxed clock coefficient of variation	ΔBF
POL (PIC1362)	Strict	Constant	-6727	0.48 (0.37 – 0.62)	NA	19.1
	Strict	Expo	-6708	0.31 (0.19 – 0.47)	NA	11.1
	Relaxed	Constant	-6699	0.68 (0.47 – 0.96)	0.89 (0.47 – 1.43)	7.8
	Relaxed	Expo	-6685	0.41 (0.29 – 0.51)	0.56 (0.32 – 0.84)	0.64
	Relaxed	BSP	-6682	0.43 (0.35 – 0.53)	0.63 (0.43 – 0.88)	0
POL (BH)	Strict	Constant	-14429	0.07 (0.04 – 0.09)	NA	24.5
	Relaxed	Constant	-14369	0.09 (0.02 – 0.17)	0.42 (0.23 – 0.73)	0
	Relaxed	Expo	-14370	0.02 (0.01 – 0.05)	0.31 (0.23 – 0.39)	0.98
	Relaxed	BSP	-14371	0.08 (0.02 – 0.13)	0.30 (0.22 – 0.39)	1.2

Table S2: **Fitted values for the WH and BH ER in each genomic region (all the datasets, see also Figure 1).** P-value indicates the outcome of a two-tailed t-test comparing WH and BH rates. Note that some values were missing for the BH dataset.

Region	Begins	Ends	Length	Overlap	$\log_{10}$(WHER)	$\log_{10}$(BHER)	p-val.
C2V5	7027	7646	619	1	-1.60	-2.46	***
ENV1	6311	8379	2068	1	-1.94	-2.52	***
ENV1-1	6311	7024	713	1	-2.04	-2.48	***
ENV1-2	7024	7380	356	1	-1.88	-2.66	***
ENV1-3	7380	7968	588	1	-1.59	-2.51	***
ENV1-4	7968	8379	411	1	-1.97	-2.90	***
ENV2	8654	8795	142	1	-1.89	-2.58	***
GAG	790	2087	1298	1	-2.76	-2.96	***
GAG-POL	2088	2292	205	2	-1.59	-2.62	***
NEF	8797	9085	289	1	-1.92	-2.73	***
NEF-LTR3	9086	9417	332	2	-2.02	-2.66	***
POL	2293	5040	2748	1	-2.55	-2.81	***
POL-VIF	5051	5096	56	2	-2.60	-3.16	***
REV-ENV	8470	8653	184	2	-1.92	missing	NA
TAT	5851	5969	119	1	-1.76	-2.55	***
TAT-REV	5970	6045	76	2	-1.80	-2.48	***
TAT-REV-ENV	8379	8469	91	3	missing	missing	NA
VIF	5097	5558	462	1	-2.35	-2.99	***
VIF-VPR	5559	5619	61	2	-3.01	missing	***
VPR	5620	5830	211	1	-2.43	-3.02	***
VPR-TAT	5831	5850	20	2	-2.40	missing	NA
VPU	6062	6224	163	1	-2.04	missing	NA
VPU-ENV	6225	6310	86	2	-2.18	-2.53	***

Table S3: **Fitted values for the WH and BH ratios in each regions (PIC1362 and US-up4 datasets).** P-value indicates the outcome of a two-tailed t-test comparing WH and BH ratios. Values in italic are not significantly different from 1 (one sample t-test).

Region	WH ratio	BH ratio	P-value
C2V5	0.90	0.97	***
ENV1	0.98	*1.00*	***
ENV1-1	0.91	*1.00*	***
ENV1-2	0.90	0.99	***
ENV1-3	0.77	*1.00*	***
ENV1-4	*0.96*	*1.00*	n.s.
ENV2	0.42	*1.01*	***
GAG	1.08	*1.01*	***
GAG-POL	0.77	0.97	***
NEF	*1.00*	0.98	n.s.
NEF-LTR	1.01	0.95	***
POL	0.96	0.99	***
REV-ENV	0.99	*1.00*	n.s.
TAT	0.92	0.95	***
TAT-REV	1.01	*1.00*	***
TAT-REV-ENV	0.92	*1.01*	***
VIF	0.99	1.02	***
VIF-VPR	0.98	0.98	n.s.
VPR	1.00	0.98	***
VPU	0.99	*1.00*	**
VPU-ENV	*1.00*	*1.01*	n.s.

References

1. Suchard MA, Weiss RE, Sinsheimer JS: **Bayesian selection of continuous-time Markov chain evolutionary models**. *Mol Biol Evol* 2001, **18**(6):1001–13.
